# Supplementary material for: A new cheese population in Penicillium roqueforti and adaptation of the five populations to their ecological niche
Source: Evol Appl. 2023 Jul 10;16(8):1438–57. doi: 10.1111/eva.13578 (PMC10445096; doi:10.1111/eva.13578)

**Supplementary Figure S1: Schematic representation of the experiments performed for testing the impact of different parameters on the growth of the five populations of the blue cheese fungus *Penicillium roqueforti*.** Growth dynamics was measured by laser nephelometry (cell density in liquid), then a primary modeling was applied to fit growth curves and estimate growth parameters (latency, growth rate and maximal growth); then, a secondary modeling was performed to estimate cardinal values (*i.e.* minimal, maximal and optimal values of the considered parameter for growth and the optimal growth, *i.e.*, the growth rate at the optimal value, corresponding to the maximal growth rate.


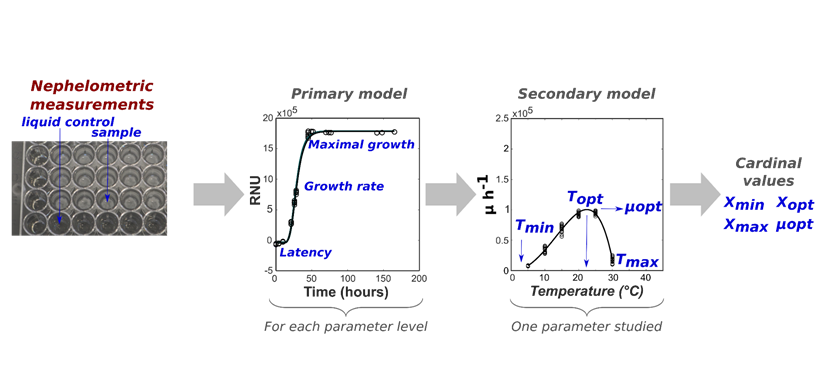

Supplement: Supplementary file 1 — Figure S1. [file EVA-16-1438-s010.docx]
