# Supplementary material for: A new cheese population in Penicillium roqueforti and adaptation of the five populations to their ecological niche
Source: Evol Appl. 2023 Jul 10;16(8):1438–57. doi: 10.1111/eva.13578 (PMC10445096; doi:10.1111/eva.13578)

**Supplementary Figure S2: Principal component analysis (PCA) based on 190,387 single nucleotide polymorphisms in the present in the five *Penicillium roqueforti* populations*:* the three cheese populations (Roquefort, non-Roquefort and Termignon) and the two non-cheese populations (silage/food spoiler and lumber/spoiled food)*.*** Dimensions 3 and 4 are shown, with their percentage of explained variation. Point in grew represent the strain unassigned to any population, ESE00421.


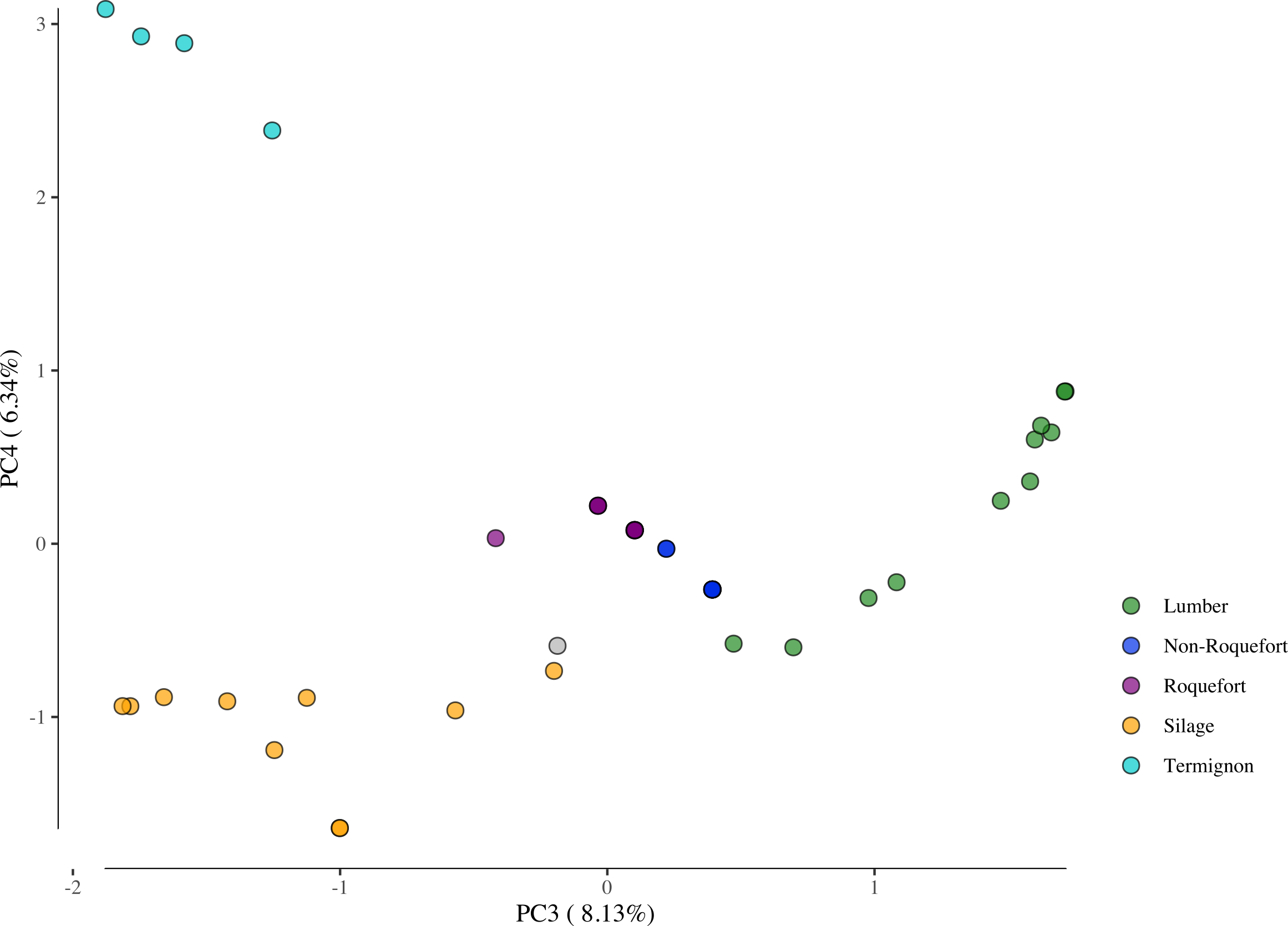

Supplement: Supplementary file 2 — Figure S2. [file EVA-16-1438-s005.docx]
