# Supplementary material for: A new cheese population in Penicillium roqueforti and adaptation of the five populations to their ecological niche
Source: Evol Appl. 2023 Jul 10;16(8):1438–57. doi: 10.1111/eva.13578 (PMC10445096; doi:10.1111/eva.13578)

**Supplementary Figure S3: Population subdivision inferred with NGSadmix. A.** Population subdivision inferred for K=2 to 6. Colored bars represent the coefficients of membership in the K gene pools based on genomic data. Each bar represents a strain, its name being indicated at the bottom of the figure. The ID of the strains used for phenotyping are in bold. The barplots represent, for each K value, the solution inferred in the highest percentage of the 100 runs and this percentage is indicated at the top. **B.** Different solutions inferred at K=6, with their percentage among the 100 runs given at the top. **C.** Second order rate of change in the likelihood (ΔK, at left) and log of the likelihood across K values.

**A**


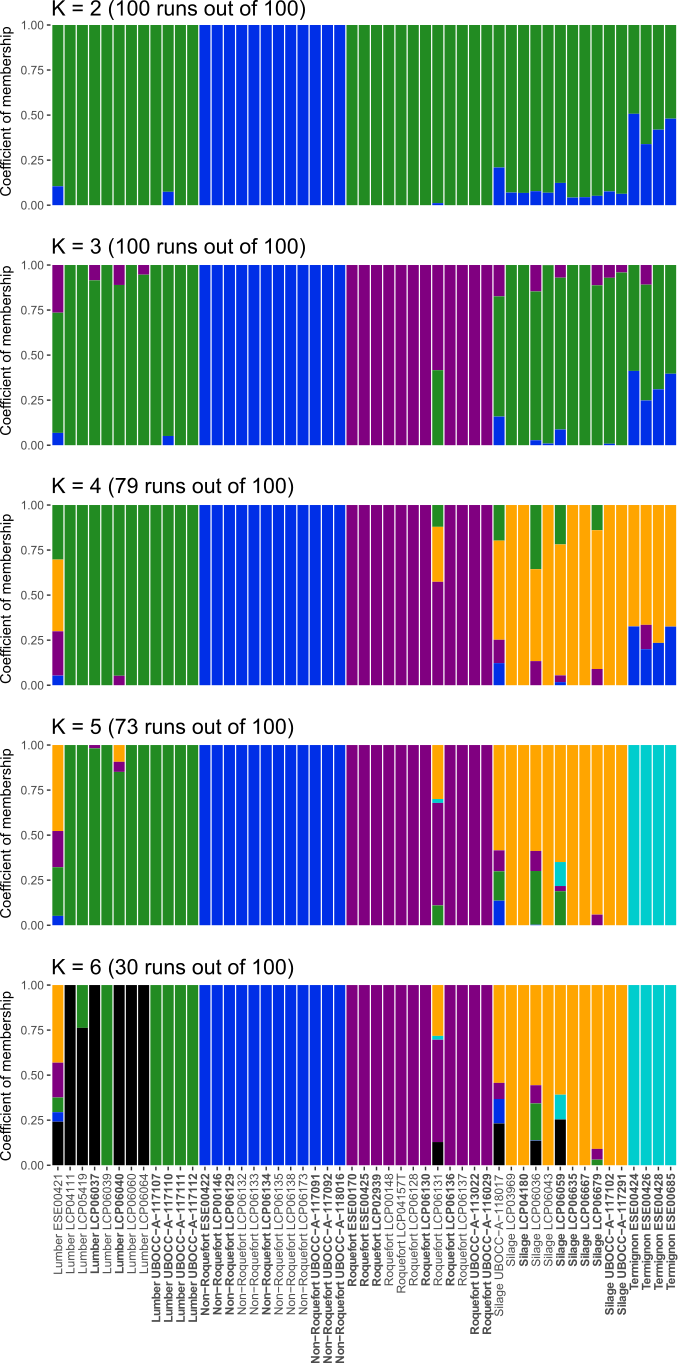


**B**


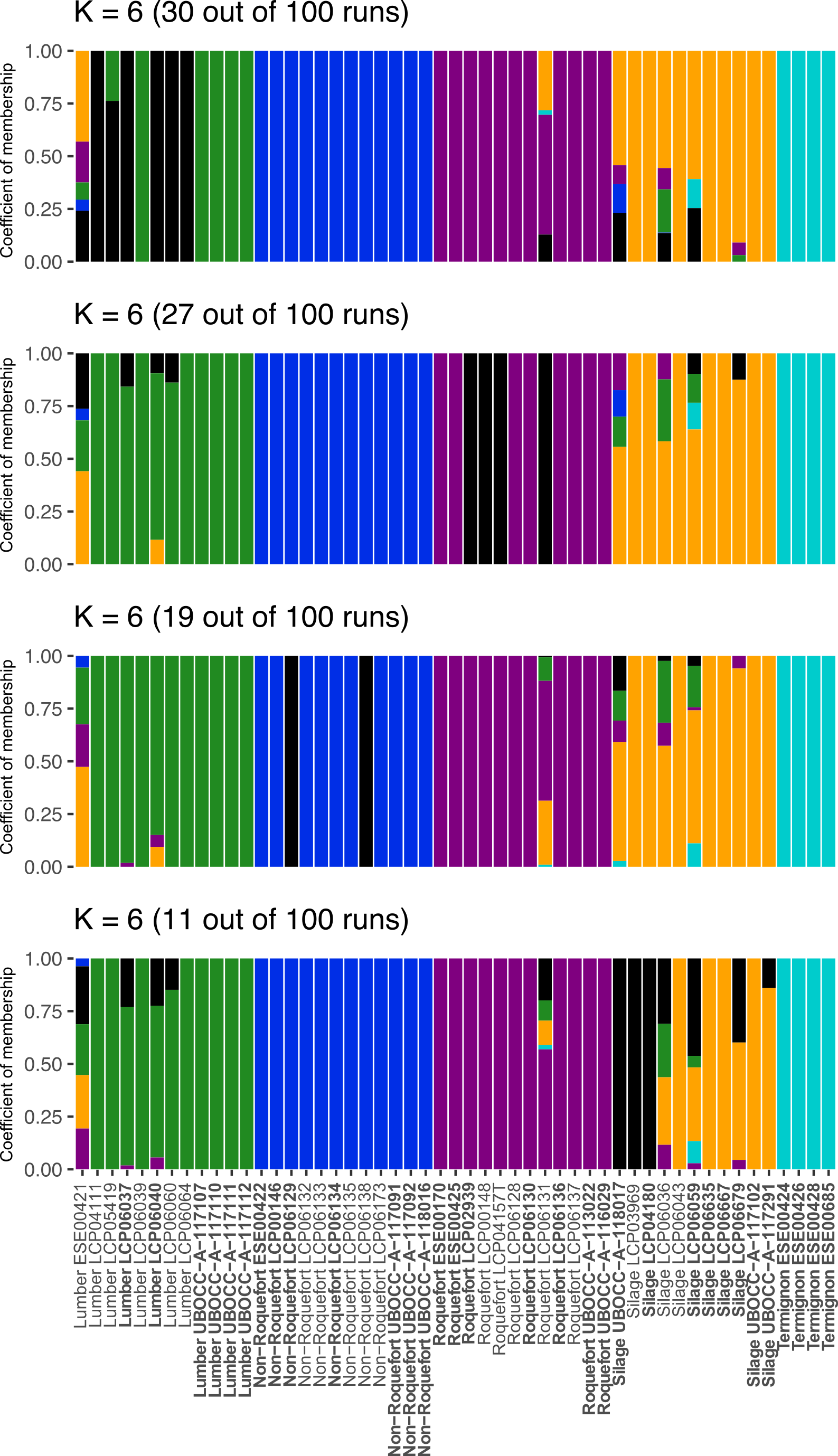


**C**


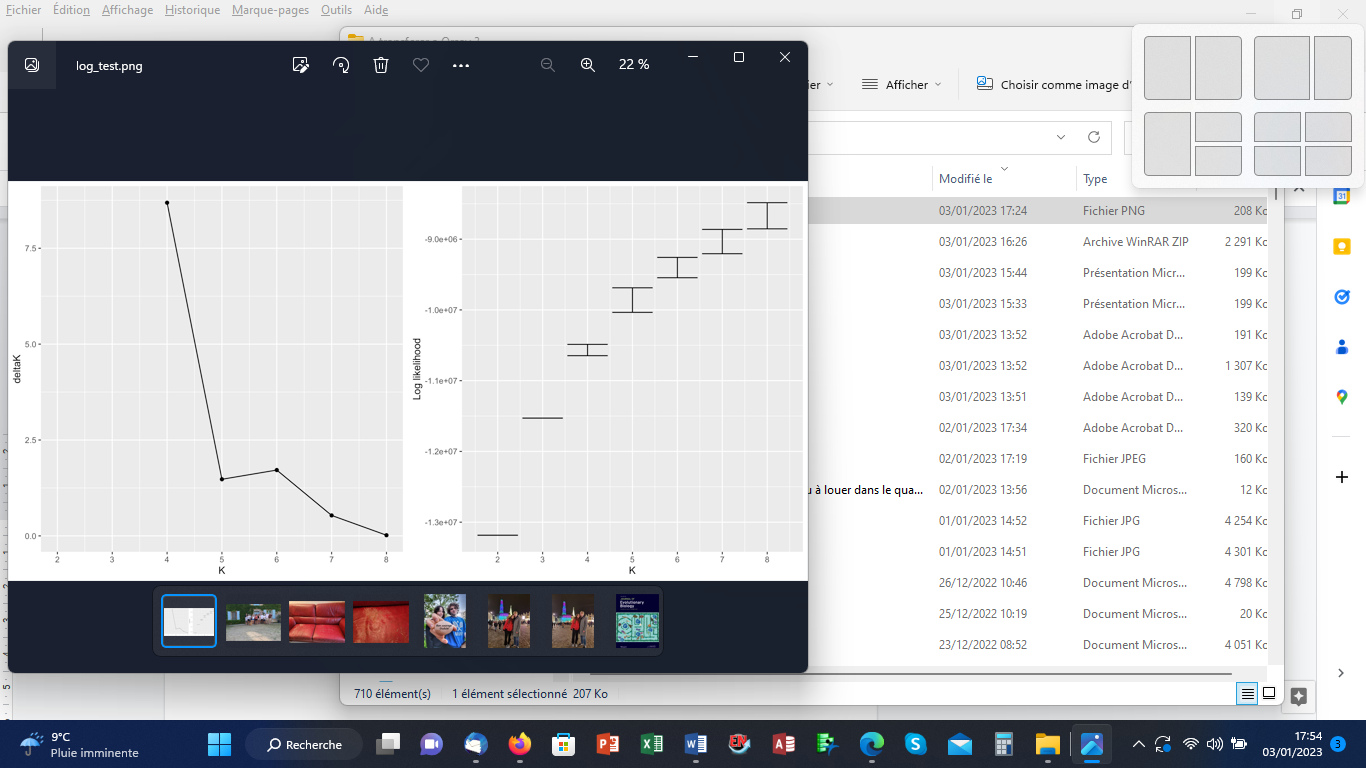

Supplement: Supplementary file 3 — Figure S3. [file EVA-16-1438-s007.docx]
