# Supplementary material for: A new cheese population in Penicillium roqueforti and adaptation of the five populations to their ecological niche
Source: Evol Appl. 2023 Jul 10;16(8):1438–57. doi: 10.1111/eva.13578 (PMC10445096; doi:10.1111/eva.13578)

**Supplementary Figure S4: Principal component analysis (PCA) illustrating the phenotypic differences between *Penicillium roqueforti* populations based on growth response to temperature, water activity (salt), pH, various carbon sources (sucrose, glucose, lactose, galactose, maltose, cellobiose, xylose, starch, pectin and lactic acid) and to exposure to fungal inhibitors (lactic acid, potassium sorbate, tebuconazole and natamycin), with dimensions 3 and 4 shown. A.** A confidence ellipse is drawn for each of the five populations. The percentage of variance explained by the axes are indicated. The same colour code is used as in the other figures: green for the lumber/spoiled food population, orange for the silage population, dark blue for the non-Roquefort cheese population, purple for the Roquefort cheese population and light blue for the Termignon cheese population. The strain IDs are provided in Suppl. Table 1. **B**. Association between the two PCA axes and the variables. Lamax.lambda.LA corresponds to maximal concentration in lactic acid compatible with growth. awmin.lambda.aw and awmin.lambda.aw correspond to minimal water activity (maximal concentration of salt compatible with growth). Teb2.5.lambda.relative and Teb5.lambda.relative correspond to latency with concentration in tebuconazole of 2.5 and 5 mg relative to latency without tebuconazole. See Suppl. Fig. S1 for an illustration of parameter determination.

**A.**


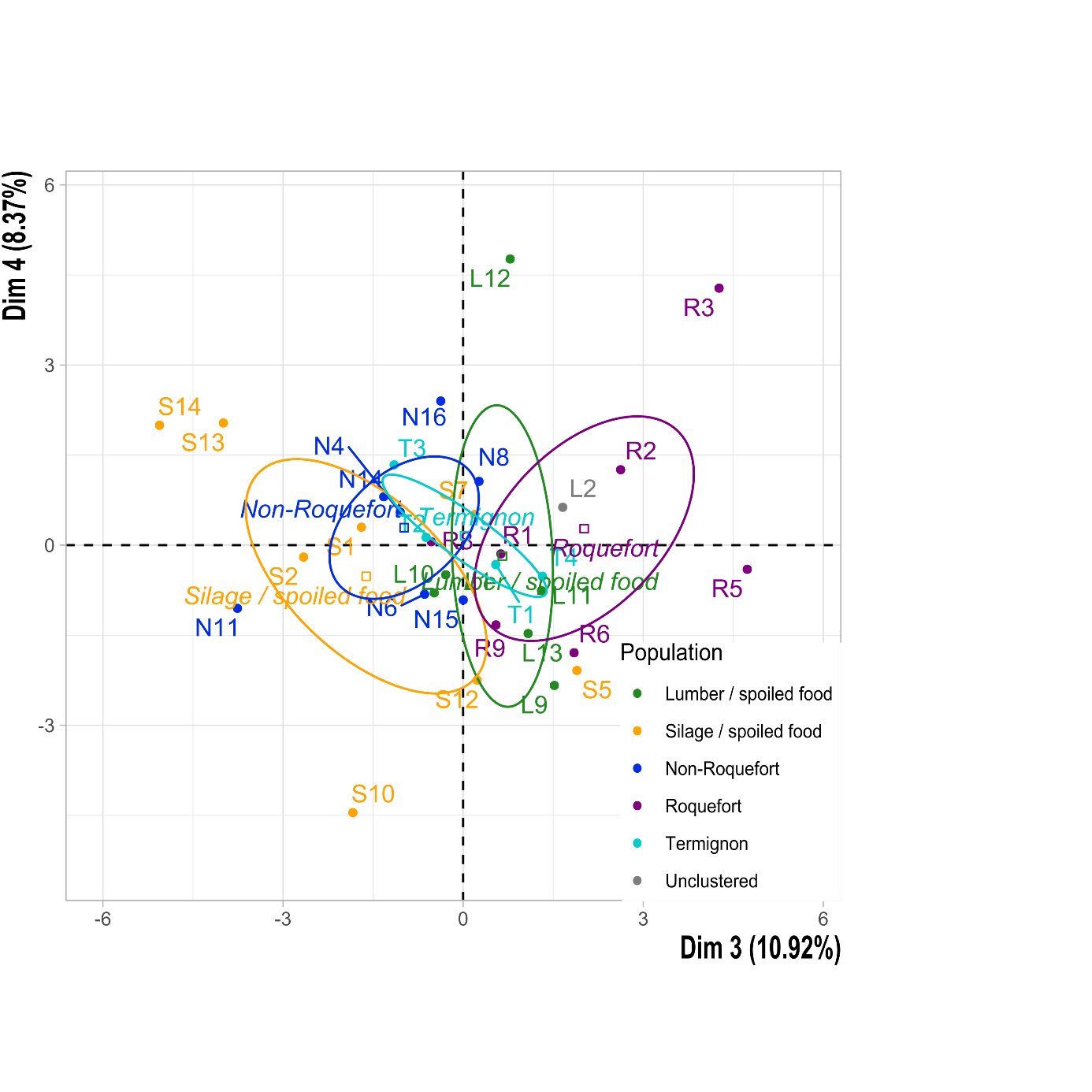


**B.**


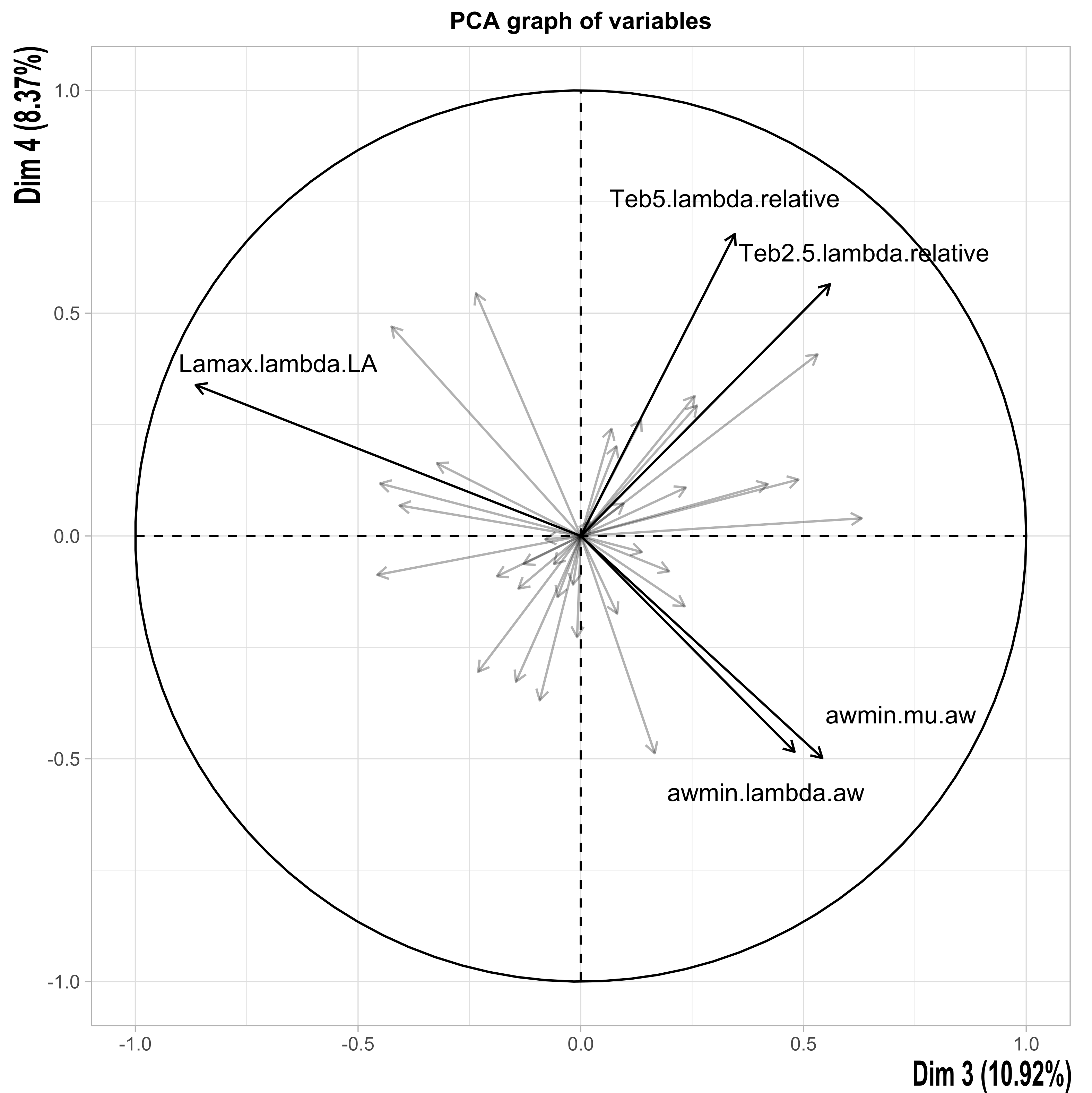

Supplement: Supplementary file 4 — Figure S4. [file EVA-16-1438-s001.docx]
